# Radiation Mapping and Laser Profiling Using a Robotic Manipulator

**DOI:** 10.3389/frobt.2020.499056

**Published:** 2020-11-26

**Authors:** Samuel R. White, David A. Megson-Smith, Kaiqiang Zhang, Dean T. Connor, Peter G. Martin, Chris Hutson, Guido Herrmann, John Dilworth, Thomas B. Scott

**Affiliations:** ^1^Department of Physics, Interface Analysis Centre, University of Bristol, Bristol, United Kingdom; ^2^Department of Mechanical Engineering, University of Bristol, Bristol, United Kingdom; ^3^Department of Electrical and Electronic Engineering, The University of Manchester, Manchester, United Kingdom; ^4^KUKA Systems UK Ltd, Halesowen, United Kingdom; ^5^RACE UKAEA, Cullham Science Centre, Abingdon, United Kingdom

**Keywords:** radiation mapping, 3D modeling, spectrometry, gamma scanning, nuclear waste, robotic manipulator

## Abstract

The use of a robotic arm manipulator as a platform for coincident radiation mapping and laser profiling of radioactive sources on a flat surface is investigated in this work. A combined scanning head, integrating a micro-gamma spectrometer and Time of Flight (ToF) sensor were moved in a raster scan pattern across the surface, autonomously undertaken by the robot arm over a 600 × 260 mm survey area. A series of radioactive sources of different emission intensities were scanned in different configurations to test the accuracy and sensitivity of the system. We demonstrate that in each test configuration the system was able to generate a centimeter accurate 3D model complete with an overlaid radiation map detailing the emitted radiation intensity and the corrected surface dose rate.

## 1. Introduction

The global nuclear industry is facing significant challenges in decommissioning and nuclear waste management owing to an ever-increasing amount of nuclear waste awaiting to be processed and prepared for long-term storage. In 2018, *Status and Trends in Spent Fuel and Radioactive Waste Management* from the IAEA Nuclear Energy Series, reported that globally there is some 6,317,000 *m*^3^ of nuclear waste in storage awaiting a long term disposal solution (IAEA, 2018). Each waste category has a different associated disposal cost, for example Low Level Waste (LLW) in the UK has an attributable cost of £2.9k per m^3^, whilst Intermediate Level Waste (ILW) has a cost of £46k per m^3^ based on 2008 data (UK Government Department of Energy & Climate Change, 2011). It may be considered logical that higher activity wastes should cost more to manage because they represent a higher hazard to humans. ILW and HLW have activities which are sufficiently extreme that humans cannot come into close contact with them, mandating their remote handling and inspection. Accordingly, on a fiscal and safety basis it is important that waste materials are not inadvertently processed into the wrong waste category. Thresholds between these waste types are clearly defined (in terms of activity per unit mass) and there is a significant cost difference in managing each waste type. Nuclear decommissioning and waste management is therefore in urgent need for technologies that deliver high accuracy and automated radiation and 3D surveying to help waste sorting. There are numerous “sort and segregation” activities which seek to characterize mixed nuclear wastes into their correct streams, both repeatedly and with a high throughput; whilst avoiding the need for cost increases through excessive conservatism (Horizon2020, 2018). There is a current funding competition call from Sellafield to address the problem of sort and segregation tables, in which technology can identify radioisotopes and objects on tables is desired to solve this problem (Gov, 2020). In addition, there is a concurrent critical need to develop and implement technologies with the ability to scan packaged wastes held in storage, to check for external radiation hot spots and/or signs of surface deformation or corrosion. In the UK, Sellafield Ltd accommodates one of the largest inventories of ILW. This must be routinely checked until such time as a Geological Disposal Facility (GDF) becomes available. An inability to routinely check waste in storage could present a multitude of issues.

An alternative approach, which is only now becoming possible, is in the use of robotic manipulators equipped with micro gamma-spectrometers to scan waste packages in a more dynamic way. Such solid-state detection units are usually very compact with detector crystals of 1–30 cm^3^ and fast counting rates (typically 20,000 cps) able to discern different gamma-emitting radioisotopes based on their differing decay energies. Conceptually, they enable radiation scans to be performed robotically at much smaller stand-off distances (<10 cm) than segmented gamma scanning, yielding a much higher spatial resolution and sensitivity. However, in order to conduct such close-proximity scanning, a method of determining the sensor stand-off distance must also be integrated. To touch the waste material could potentially contaminate or damage the detector and hence this needs to be prevented at all costs.

There are therefore numerous robotic technologies and sensors which are capable of being combined to achieve this target of combined gamma scanning and 3D profiling of nuclear waste objects—yet to our knowledge this has not previously been reported. The use of robotics for gamma inspection in the nuclear industry is not new, but has typically been employed for plant inspection and not waste assay. Tsitsimpelis et al. (2019) discuss further developments in a recent review paper on ground-based robotic systems for the characterization of nuclear environments, highlighting numerous robotic systems which have been deployed for radiation monitoring within the last 50 years. In 1994, Redus et al. (1994) published a paper on the use of video and gamma ray imaging systems for inspection robots in nuclear environments. The group used a robot with a mounted gamma spectrometer and camera to record video footage with super-imposed gamma ray imaging enabling the identification of radioactive sources in a room. This is the first example of a robotic radiation mapping procedure. Since then Bird et al. (2019) have developed this concept by researching the use of mobile robotic platforms for the routine inspection of nuclear facilities. The Continuous Autonomous Radiation Monitoring Assistance (CARMA) robot uses LiDAR sensors for Simultaneous Localisation and Mapping (SLAM), generating a 2D plan map of the room. In addition to this, they obtain gamma radiation intensity mapping data from a Thermo Fisher Scientific RadEye. In a similar way, a considerable amount of radiation mapping research has been conducted with the use of Unmaned Aerial Vehicles (UAVs) to map radiation at nuclear sites including the Chernobyl and Fukushima fallout zones. Martin et al. (2017) presented “High-Resolution Aerial Radiation Mapping for Nuclear Decontamination and Decommissioning”. The UAV flew autonomously along GPS defined flight paths above the Sellafield nuclear decommissioning facility, using a sensor package which simultaneously recorded GPS position, above ground height using a ranging LiDAR and gamma spectrometry data. This data was used to produce an accurate radiation map of each survey area studied, as well as using the spectral information from gamma measurements data to identify various radioactive isotopes in different facilities (Martin et al., 2016b). All of these techniques connect radiometric data to positional data to generate a radiation map.

The use of point cloud data in the formation of 3D models is another recent innovation that the field of robotics has been quick to adopt. Various techniques are routinely being applied to generate point cloud data, which in turn can be transformed into a 3D model. Within nuclear robotics, LiDAR scanning is already an established technique used in 3D environment reconstruction. Aerial radiation mapping routinely relates recorded radiometric data to a 3D model collected by either LiDAR or photogrammetry to produce a combined 3D representation (Connor et al., 2016; Martin et al., 2016a). There are numerous examples of high quality 3D models being generated for robotic systems (Marturi et al., 2018; Sarker et al., 2019; Barone et al., 2020). However they are all reliant on Charged Coupled Device (CCD) cameras. The main issue here is that within a highly radioactive environment CCD based devices fail, as the gamma radiation causes damage to their internal Metal Oxide Semi-conductor (MOS) capacitors. More novel are Time of Flight (ToF) scanners and cameras that are often able to generate data from complex objects for 3D reconstruction. The advantage here is ToF sensors often have small size and therefore small gamma interaction cross sections, making them more radiation hard. Hoegg et al. (2013) managed to reconstruct 3D models of a selection of cars using a ToF camera, whilst Gutierrez-Villalobos et al. (2017) created an accurate 3D model of a plastic cup using a cheap off the shelf VL53L0X ToF scanner. In this latter work, a VL53L0X was located in a fixed position and a plastic cup rotated and moved vertically in front of it. By recording the position of the cup relative to the detector and building cloud point data, the team successfully generated a 3D model of the cup.

The research in the current manuscript adopts a similar process to Martin et al. (2017) to perform close scanning of simulated nuclear waste but using a robot arm as the mobile platform. We use the co-ordinates generated by a robotic arm, combined with ToF ranging at the front of the sensor to determine its position relative to the target objects. The ToF sensor is for exact stand-off distance measurement to permit accurate radiation dose conversions. Other standard techniques could be used to make a higher resolution 3D model, for example photogrammetry or 3D lidar, from a greater distance where dose is lower. This novel scanning work builds toward the end goal of more accurately scanning mixed wastes produced during decommissioning as well as existing packages for routine assay. The use of industrial robots, such as those made by KUKA, is well-understood and many are already in use on nuclear sites around the world. The high spatial precision and repeatability of KUKA manipulators means that they can be accurate at the sub-millimeter scale, making them an ideal candidate platform for high precision detector research.

## 2. Experimental Setup

**Figure 1:**
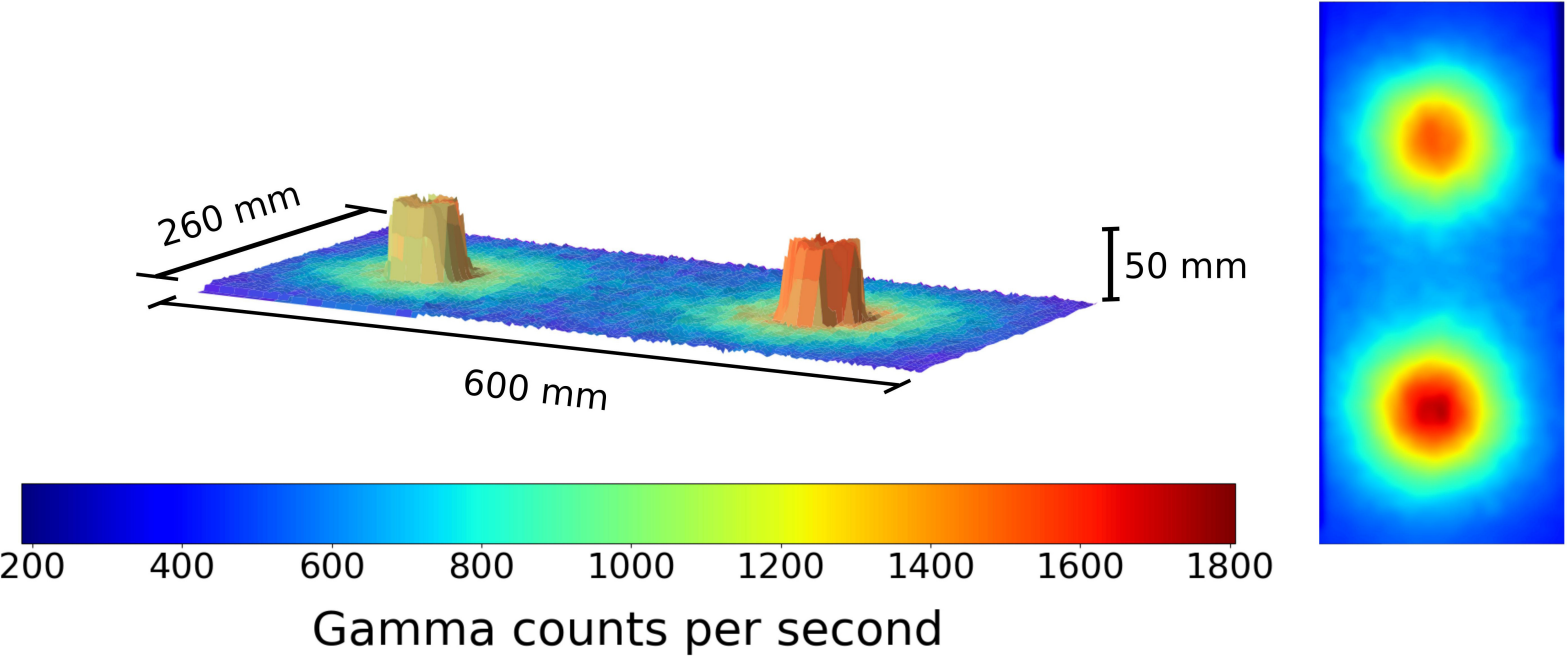
A figure showing the 3D model and overlaid radiation map generated when the robot scan was completed at a 1 cm standoff above 2 Cs-137 sources 30 cm apart. Left to right the source activity is 7.5 and 10 μSvh^−1^, respectively. The color represents the gamma radiation counts in counts per second for a given 2 × 2 mm pixel.

**Figure 2:**
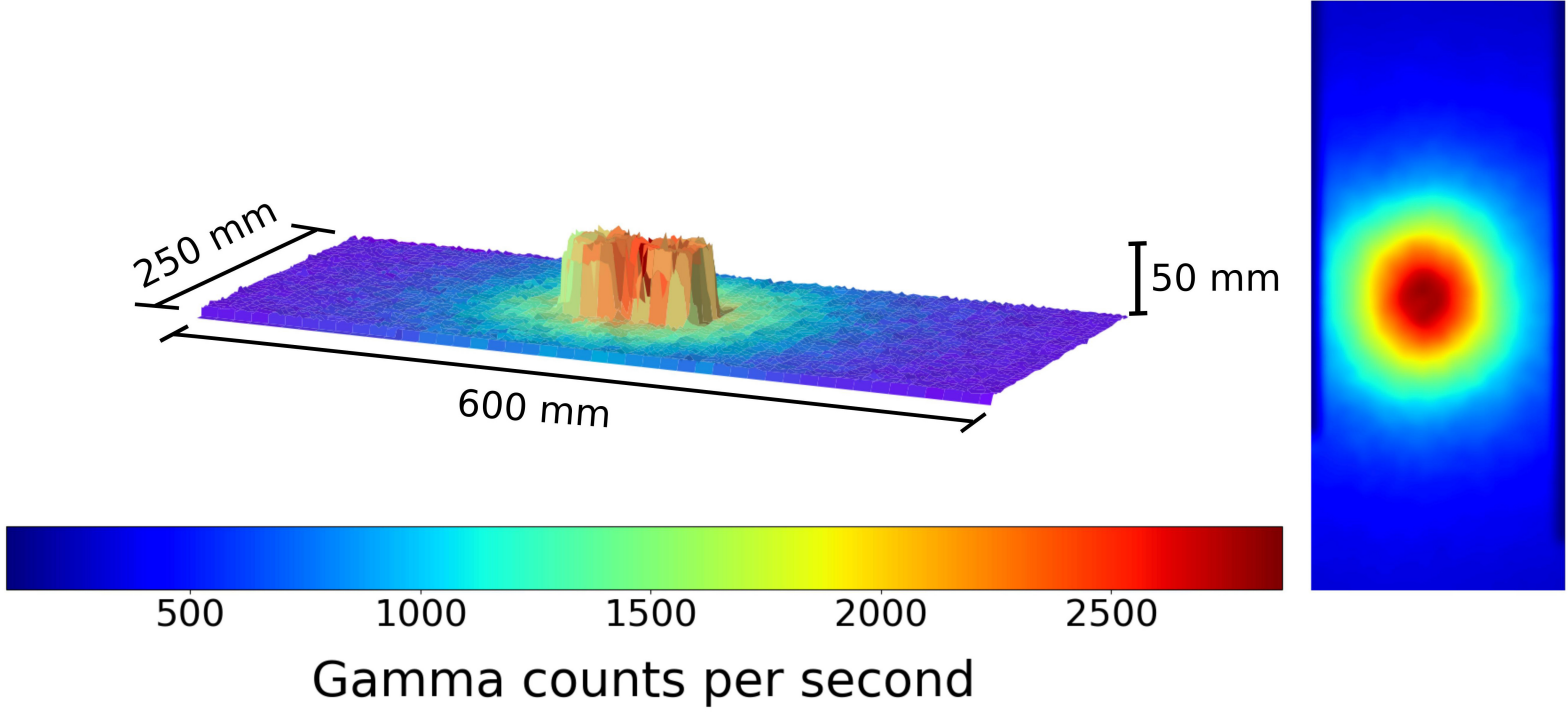
A figure showing the 3D model and overlaid radiation map generated when the robot scan was completed at a 1 cm standoff above 2 Cs-137 sources directly adjacent. Left to right the source activity is 7.5 and 10 μSvh^−1^, respectively. The color represents the gamma radiation counts in counts per second for a given 2 × 2 mm pixel.

**Figure 3:**
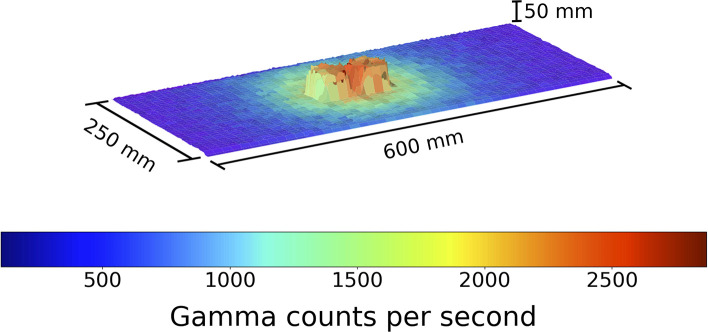
A figure showing the 3D model and overlaid radiation map generated when the robot scan was completed at a 1 cm standoff above 2 Cs-137 sources directly adjacent, from a second angle to aid visual clarity of the distinguished pucks. Left to right the source activity is 7.5 and 10 μSvh^−1^ respectively. The color represents the gamma radiation counts in counts per second for a given 2 × 2 mm pixel.

**Figure 4:**
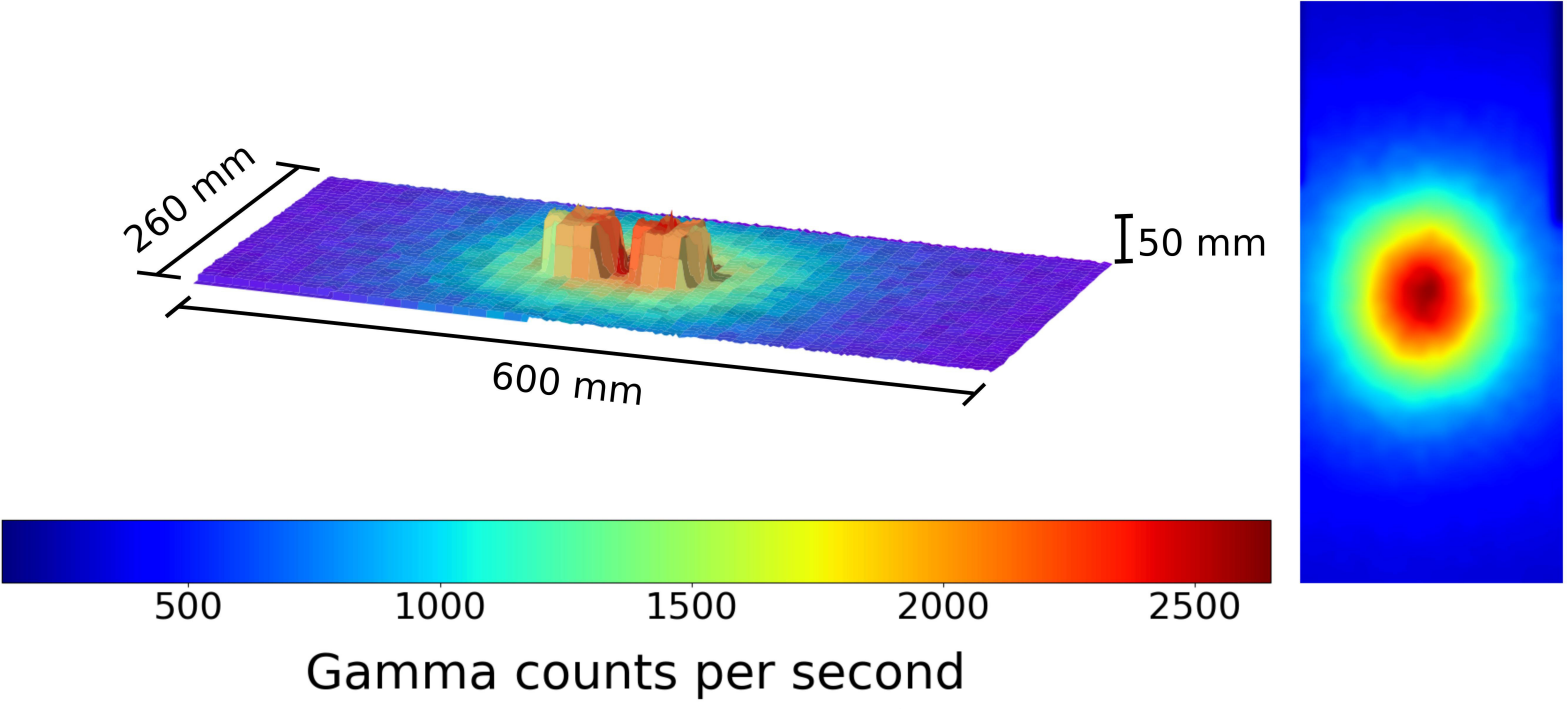
A figure showing the 3D model and overlaid radiation map generated when the robot scan was completed at a 1 cm standoff above 2 Cs-137 sources 1 cm apart. Left to right the source activity is 7.5 and 10 μSvh^−1^, respectively. The color represents the gamma radiation counts in counts per second for a given 2 × 2 mm pixel.

**Figure 5:**
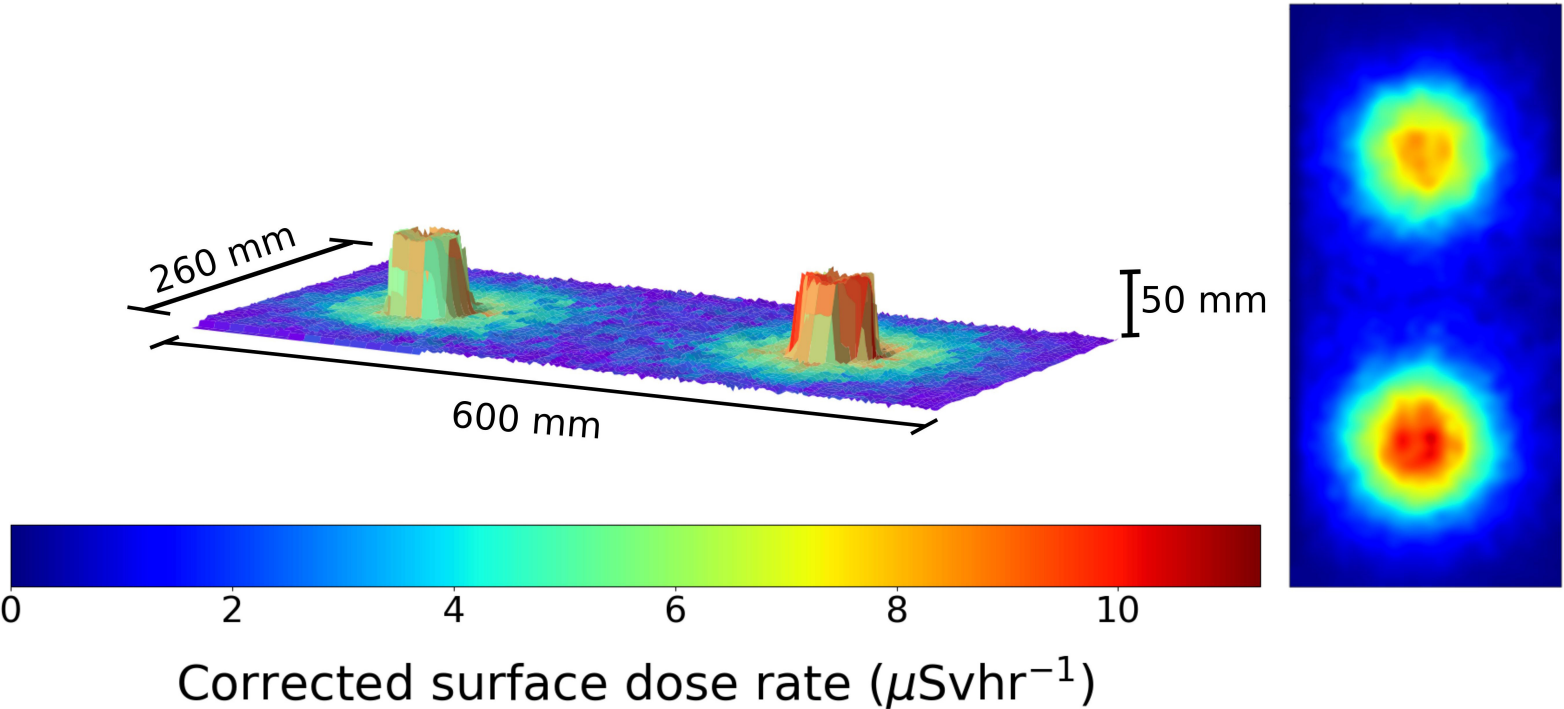
A figure showing the 3D model and overlaid radiation map generated when the robot scan was completed at a 1 cm standoff above 2 Cs-137 sources 30 cm apart. Left to right the source activity is 7.5 and 10 μSvh^−1^, respectively. The color represents the radiation dose rate in μSvh^−1^ for a given 2 × 2 mm pixel.

**Figure 6:**
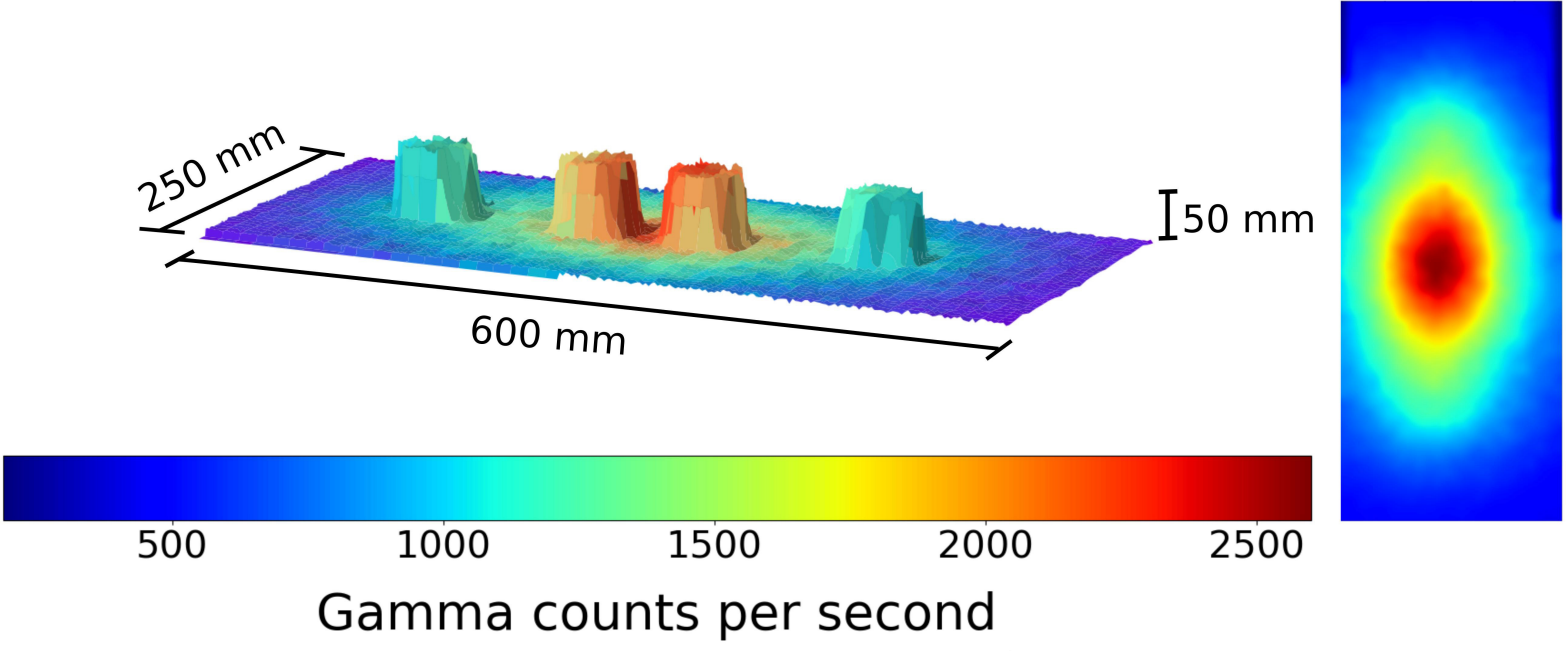
A figure showing the 3D model and overlaid radiation map generated when the robot scan was completed at a 1 cm standoff above 2 Cs-137 sources 1 cm apart and 2 Pitchblende sources 30 cm apart. Left to right the source activity is 4.5 μSvh^−1^ Pitchblende, 7.5 μSvh^−1^ Cs-137, 10 μSvh^−1^ Cs-137 and 4.3 μSvh^−1^, respectively. The color represents the gamma radiation counts in counts per second for a given 2 × 2 mm pixel.

**Figure 7:**
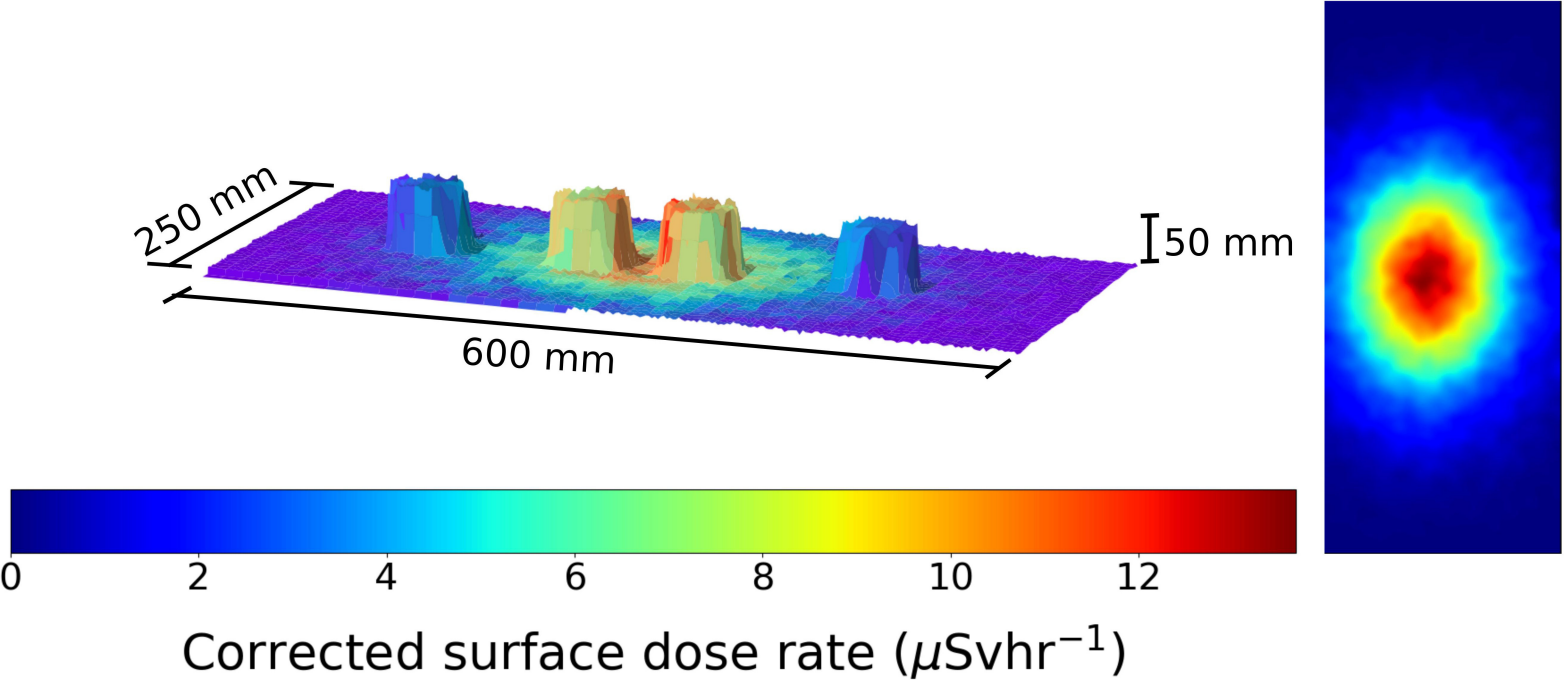
A figure showing the 3D model and overlaid radiation map generated when the robot scan was completed at a 1 cm standoff above 2 Cs-137 sources 1 cm apart and 2 Pitchblende sources 30 cm apart. Left to right the source activity is 4.5 μSvh^−1^ Pitchblende, 7.5 μSvh^−1^ Cs-137, 10 μSvh^−1^ Cs-137, and 4.3 μSvh^−1^, respectively. The color represents the radiation dose rate in μSvh^−1^ for a given 2 × 2 mm pixel.

**Figure 8:**
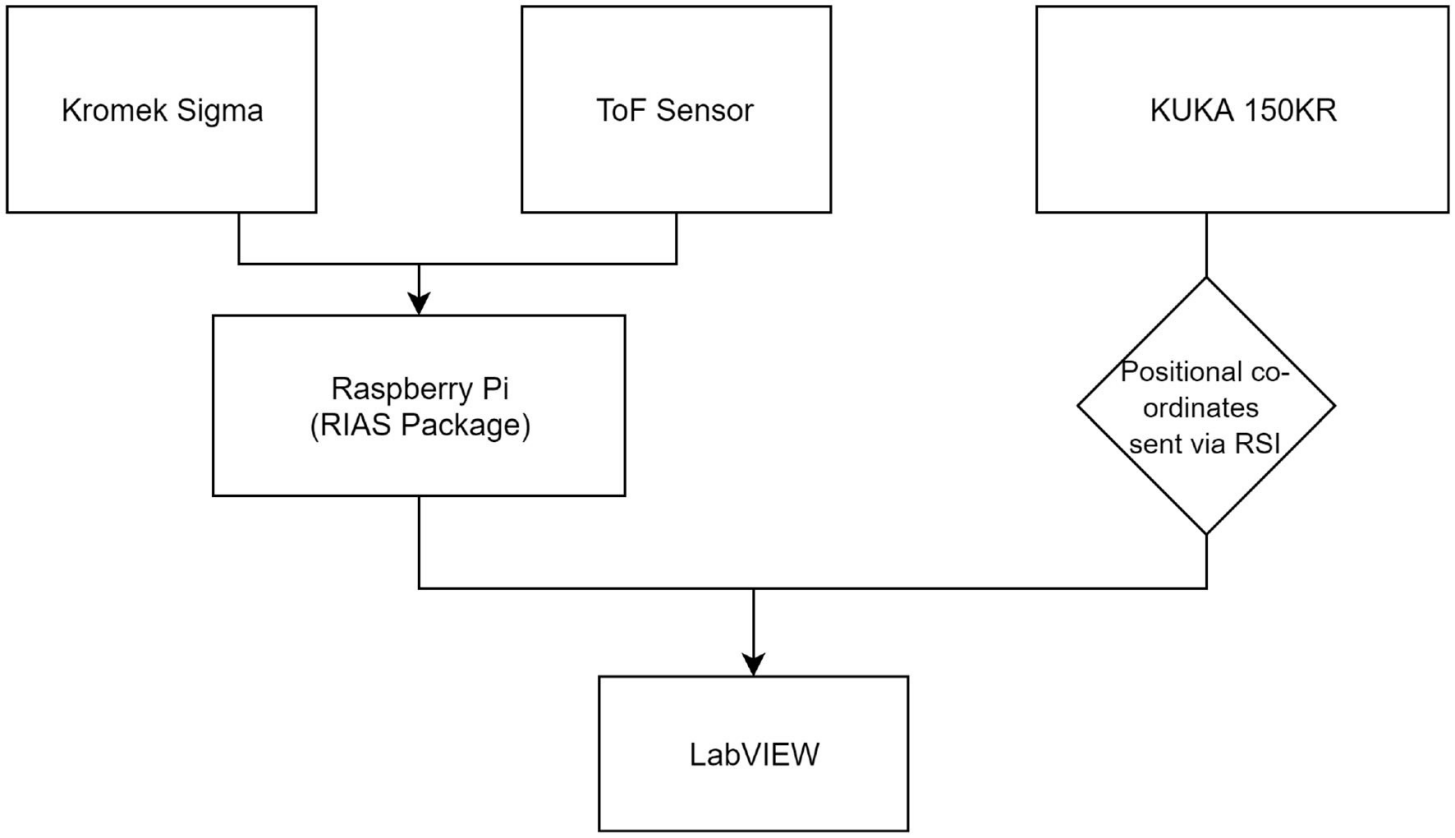
A flowchart showing how the system was integrated.

Robotic manipulation and instrument scanning must be well-synchronized to provide effective integrated measurements. For radiation scanning a Kromek^*TM*^ Sigma was utilized. The Sigma incorporates a Thallium doped Caesium Iodide (CsI(TI)) scintillator crystal, 25 × 25 × 50 mm (Kromek, 2019), contained inside a 1 mm thick aluminum casing. The maximum count rate of the Sigma is 5,000 counts per second (CPS) (Connor et al., 2018) recording gamma photons over a 50 keV to 2 MeV energy range (Kromek, 2019). It operates effectively at room temperature and does not require active cooling, unlike other more classical semiconductor detectors such as HPGe or Si (Cherry et al., 2012); this makes it an ideal choice for this application. A lead (Pb) collimator was designed to surround the detector, to reduce as far as possible the higher angle extraneous gamma counts incident on the detector. A square opening on the front face of the collimator enabled the counts to be received from a limited solid angle, perpendicular to the scan surface. The radiation detection software was programmed on a Raspberry Pi by ImiTec Ltd as part of their Remote Isotopic Analysis System (RIAS) and recorded the total number of counts received within a given exposure time. This was sent via a server communication to LabVIEW across 4096 energy bins which could request detector data at various time intervals (typically 1 or 10 Hz). The system was programmed to receive total counts and spectral data every 100 ms. Using the Robot Sensor Interface (RSI) software provided by KUKA robotics, a 6 figure co-ordinate detailing the position of the robot flange (end piece of the robot) was attained with a time stamp. The orientation of the end-flange was fixed, such that it was constantly parallel with the work surface. The two readings were time stamped and synchronized within the LabVIEW software. The LabVIEW code generated a CSV file which included the x-y position of each measurement location on the table, the distance to the point from the robot arm (from the ToF sensor) and the number of gamma counts collected. This CSV file was then processed by a python script to interpolate the data into a 3D radiation map. The data was approximated to a series of points, with 2 mm lateral spacings using a linear interpolation. These 2D points were given a third dimension by using the ToF readings. The ToF points were similarly interpolated and the resulting data is displayed in Figures 1–4, 5–7. The data collection process is shown in a flowchart in Figure 8. A VL53L0X time of flight (ToF) sensor was used to collect point cloud data to visualize the data in 3D. The ToF sensor transmits infrared light to measure distance. The solid angle produced by the stock ToF sensor is around 45 degrees, consequently it was found to regularly return anomalous data in testing, in particular where surface topography changes were more drastic. Hence a 3 mm collimating ball lens was fitted to the VL53L0X for this project, to collimate the beam and increase the spatial resolution of the scan. The radiation response of the detector was predicted to be good, as it has a small interaction cross section of around 0.25 cm^2^. The time of flight scanner takes numerous measurements of the scene whilst moving on the arm. It was mounted on the scanning head, remaining perpendicular to the table at all times. The detector was longer in one axis, giving it greater sensitivity in one dimension. This dimension was kept perpendicular to the scan table at all times to ensure a consistent radiation map was generated at the highest resolution possible. For each 100 ms exposure time, the average reading collected from the sensor was returned via the LabVIEW code.

The data collected by the arm contained a full spectrum of 4096 energy bins ranging from 50 keV to 2 MeV. This allows for the distinct Cs-137 peak at 662 keV to be identified. A python script was written to calculate the counts identified within the peak range and subtract from that the baseline reading to correct for background. A multiplication factor was applied to convert the raw count value within the energy range to a dose rate in μSvh^−1^, using the method described in Connor et al. (2020).

**Figure 9:**
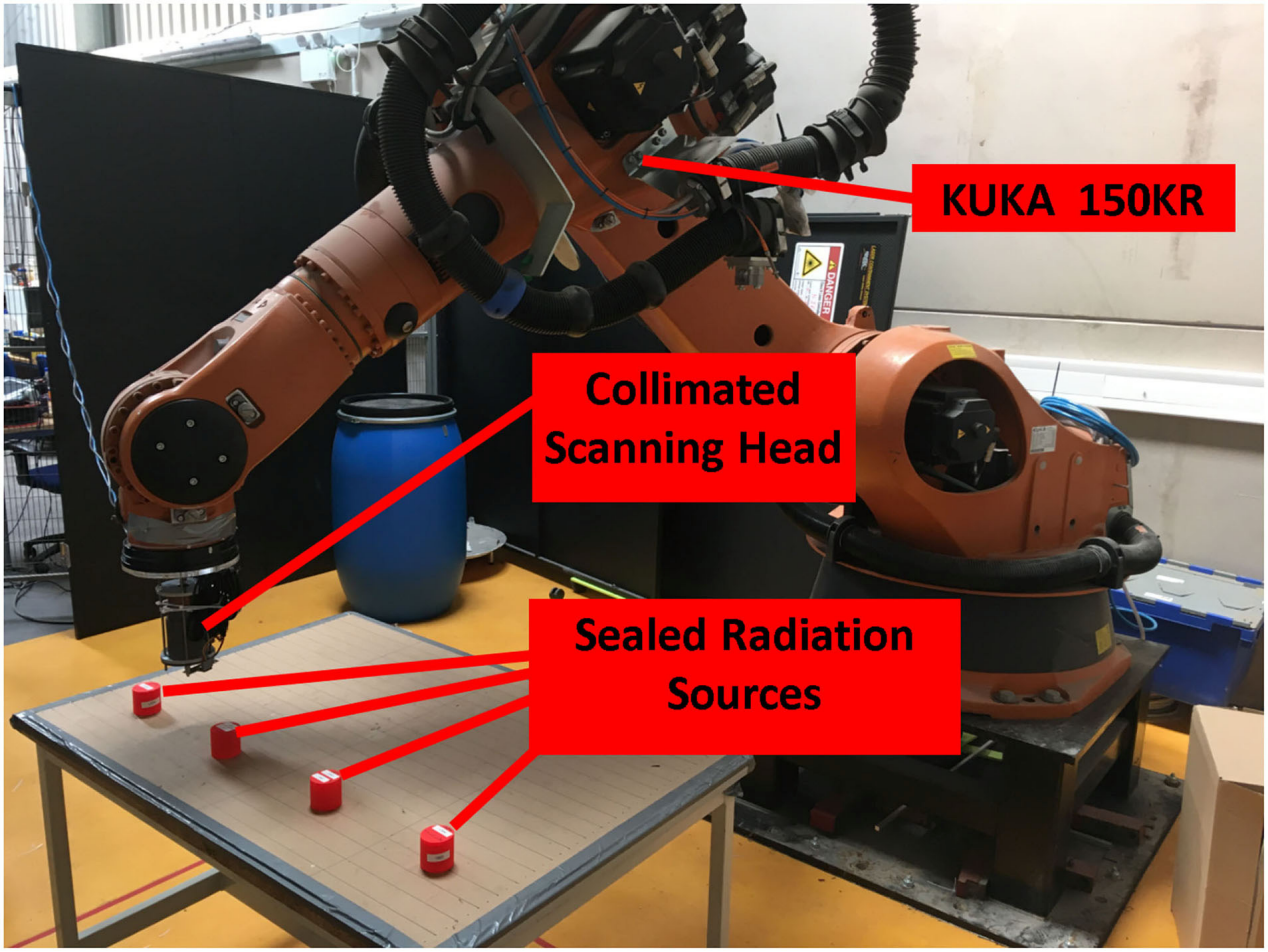
A photograph of the scanning system in action.

For scanning tests the robot was programmed to perform a basic raster pattern scan of the “scan surface”, which was a 0.6 × 0.26 m area. The raster scan had a step length of 1 cm and the speed of the scan could be varied, depending on the activity of the test sources, less active sources require longer counting times (slower scans) to achieve adequate detection. Sealed radioactive sources containing caesium-137 (Cs-137), one of 7.5 μSvh^−1^ contact dose and the other at 10 μSvh^−1^ contact dose were used, alongside naturally occurring uranium (pitchblende) sealed sources of 4.3 and 4.5 μSvh^−1^ contact dose rate to test the radiation response of the system. The robot arm was set up to move at a consistent speed in a continuous linear motion of 10 mm per second, with a single scan taking approximately 30 min. Scan time could be reduced if higher activity sources were used, but as a proof of concept where timing is not restricted, greater scan time is able to yield a higher resolution radiation map, with a longer exposure for each collection interval. A photograph of the scanning system is shown in Figure 9.

## 3. Results

To test the system, several different scanning scenarios were set up using the Cs-137 and Pitchblende sources available. The first used 2 Cs-137 sources separated apart by a distance of 30 cm, center to center. The scanning head was programmed to trace the raster scan path designed at a rate 10 mm per second. The resulting data can be seen in Figure 1.

This result demonstrates the capability of the 3D model generation using the ToF sensor, as it generates an identifiable geometric representation of the source pucks. The data is displayed with the counts recorded by the detector for a given 100 ms exposure. Following this experiment, the two sources were placed directly next to each other. This served as a test of the ToF mapping procedure. The resulting figure is shown in Figure 2.

From this we can clearly identify the radioactive hot spot. In addition the physical 3D separation of the sources can be comprehended. It is easier to distinguish on software which enables the rotation of the generated model. In order to aid the visual clarity in distinguishing the pucks, Figure 3 presents the same data in Figure 2, but from a new observation angle.

To further monitor this 3D modeling routine, the sources were next placed 1 cm apart from each other. The result of this test is shown in Figure 4.

**Figure 10:**
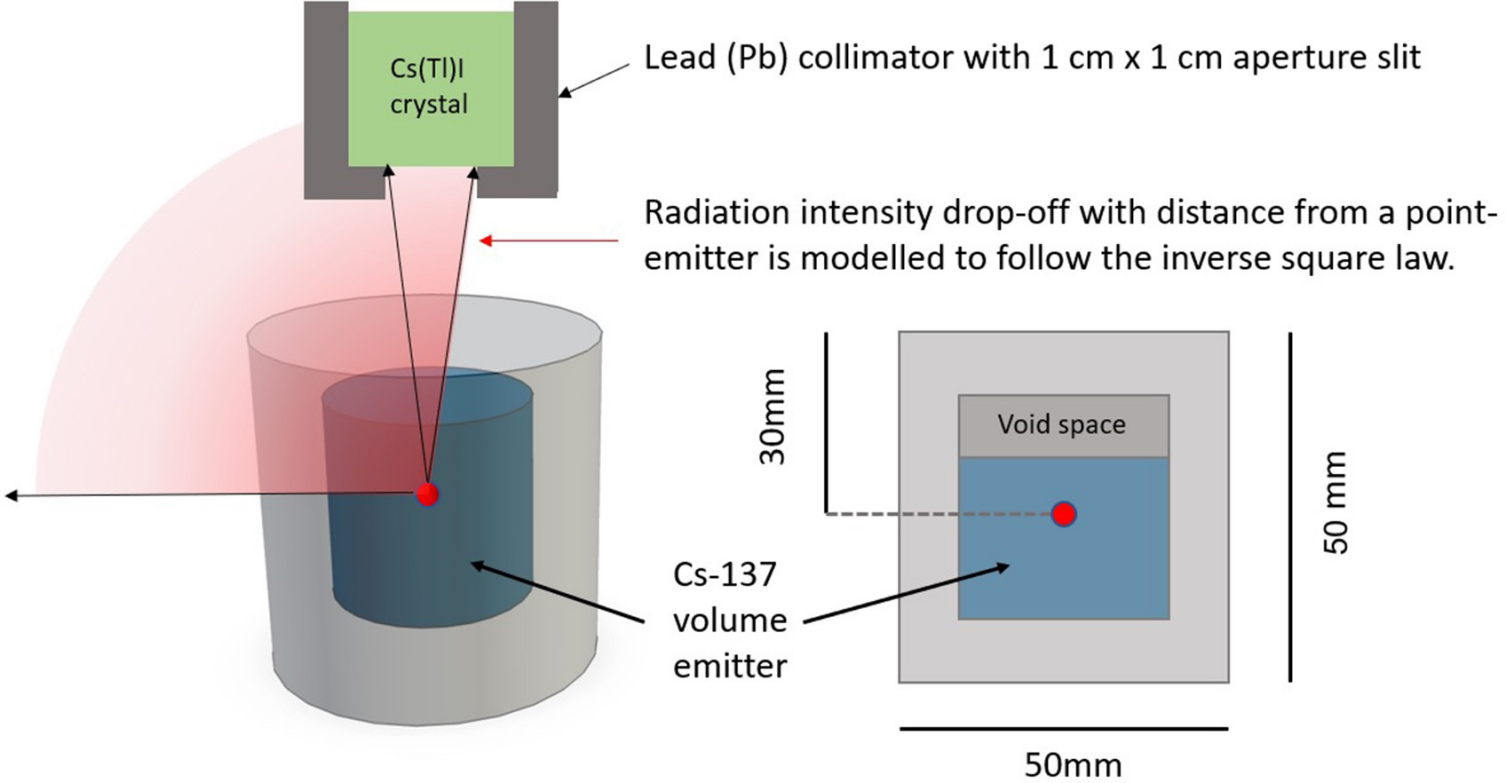
A diagram explaining the point source modeling used to invoke the inverse square law.

Here the centimeter gap is visible and both sources may be physically distinguished. This concept works and is sufficient for identifying radiation hotspots present in given scan and sort scenarios. However it does not provide any dose rate information. The data was subsequently processed to give an estimate of the dose rate of the given sources. The sources comprised of Cs-137 containing moss samples collected from Fukushima and were hence not perfect point-source emitters. Instead we approximated each source as a point emitter where the origin of the point was 3 cm beneath the puck surface, which corresponds to the thickness of the perspex and a small air gap, as shown in Figure 10.

For our calculations we assume that the perspex is gamma transparent due to its low density and Z number. Hence we may apply a correction factor to the dose rate method described in the above section, to display the data in surface dose rate format. The two Cs-137 sources positioned at 30 cm apart may be seen on a dose rate map in Figure 5.

The dose rates recorded at the 1 cm standoff are in good agreement with the actual recorded contact dose rates measured for each source: 7.5 and 10 μSvh^−1^. This is very promising in demonstrating that the system sensitivity is sufficient to discriminate radioactive objects that classify at the Very Low Level Waste (VLLW) to Low Level Waste (LLW) threshold. In the UK VLLW is normally regarded as material with a specific activity up to 100 Bqg^−1^ (RWM, 2010).

An ability to measure the full gamma radiation spectrum enables different radioisotopes to be identified. Figure 6 shows the radiation intensity map of 2 NORM pitchblende sources and two Cs-137 sources.

One can determine they are all radioactive, with different intensities, but not tell which source is which. By restricting the spectral window to only the 662 keV gamma photons, one generates, as a Cs-137 specific plot, as in Figure 7.

Using spectral gating like this the Pitchblende sources become invisible to the radiation scanning system. This is key for the nuclear industry, as it would enable radionuclide characterization and separation to be carried out autonomously.

## 4. Discussion

This paper introduces a novel integration of technologies that facilitates the scanning of radioactive materials and waste-forms, creating a 3D model of the object or environment and adding an overlaid radiation map. The setup is comprised of certified commercial off-the-shelf (COTS) components that in consequence require little control performance verification. Each component is integrated in a modular manner system, allowing for a highly flexible system design. The nature of the integrated system means that a multitude of sensor packages and grippers could be added alongside or in place of the combined detector unit used in this paper. Even the robotic arm used for this project could be replaced with an alternate choice as there are numerous different robotic arm systems commercially available of different sizes, lift capabilities, reaches and radiation tolerances that could enable a range of different scale applications, from sorting bulk rubble or pipework to sifting sediments for hot micro-particles.

Our experiments demonstrate that it is possible for such a scanning system to make very accurate, high sensitivity, high spatial resolution radiation maps for resolving nuclear waste materials from each other on the basis of emitted gamma intensity. The result was also successful from a 3D modeling perspective, as it clearly identifies the sources as separate objects despite their close proximity. The spherically symmetric radiative flux which is emitted from the radioactive sources means that there are limitations on identifying which physical shape corresponds to which emission on the radiation map. This is something that could be improved by an algebraic reconstruction technique and a comprehensive understanding of the detectors response. This forms an important part of the future work this project will require. The radiation sources used in this work were relatively weak compared to real ILW and LLW. For real waste scenarios it would be expected that a smaller micro gamma spectrometer with greater peak dose measurement capability e.g., CZT or GaAs, could be utilized. Scan times would also be dramatically reduced with increasing radioactivity levels.

The next proposed step in development is to use the laser profiling to generate a volume for the object and then based on an assumed density, e.g., for concrete, the system could be developed to automatically threshold objects and assign them as either VLLW, LLW, or ILW based on their radioactivity and calculated mass. This thresholding programme would need to utilize an algorithm based on the inverse square law to calculate the intensity of the emitted radiation at the surface of the objects being scanned, accounting for the efficiency and solid angle of the radiation detector being used. It is crucial to get an accurate distance from the detector to the target object because contact dose calculations are based on the inverse square law. This means any discrepancy in reading will significantly affect the corrected dose calculation. Ultimately, the full gamma spectrometry capability of the system could be used to distinguish different gamma emitters too. This would add a further level of finesse for separating mixed nuclear wastes that might be expected to arise during nuclear decommissioning activities. The accuracy of the 3D model would facilitate a robotic manipulator for grasping in addition, fulfilling the requirements of the sort and segregation table.

The system's scanning methodology could also be substantially refined vs. the current simple raster scanning we have demonstrated. For example, an initial survey scan could conduct rapid raster scan of a scene to determine the degree of variability in scene topology and from that, determine automatically a more detailed scanning path that would maintain a safe but close scanning proximity to the waste objects. The initial survey scan would also locate any strong radiation emitters, which the adaptive path plan could deferentially focus on to provide a more detailed scan of that specific area of the scene.

## 5. Conclusion

This work demonstrates the use of a combined laser profiling and gamma-scanning sensor unit, mounted on a robot arm, to form an accurate 3D profile of a series of test objects on a scanning table, with a coincident overlay of the mapped radiation intensity. Radiation maps are successfully created by the system, which is able to correctly identify radioactive sources of different intensities on a flat scan surface. The generated 3D surface model reveals an accurate visualization of the tested scene and is accurate to within a centimeter. In addition to this an estimate of the surface dose rate produced by the radioactive emitters is made based on the scan data received to a good level of accuracy, correctly identifying the dose rate of two radioactive Cs sources to within 1 μSvh^−1^.

## Data Availability Statement

The raw (unprocessed) data that supports the findings from this study are available from Mendeley Data with the link: doi: 10.17632/gts8x9f84r.1.

## Author Contributions

All authors contributed technically, both in the data collection and writing of this paper.

## Conflict of Interest

JD was employed by the company KUKA Systems UK Ltd. The remaining authors declare that the research was conducted in the absence of any commercial or financial relationships that could be construed as a potential conflict of interest.
